# Engineering *Saccharomyces cerevisiae* with the deletion of endogenous glucosidases for the production of flavonoid glucosides

**DOI:** 10.1186/s12934-016-0535-2

**Published:** 2016-08-04

**Authors:** Huimin Wang, Yan Yang, Lin Lin, Wenlong Zhou, Minzhi Liu, Kedi Cheng, Wei Wang

**Affiliations:** 1 State Key Laboratory of Bioactive Substance and Function of Natural Medicines, Institute of Materia Medica, Peking Union Medical College and Chinese Academy of Medical Sciences, 1 Xian Nong Tan St., Beijing, 100050 China; 2 Key Laboratory of Biosynthesis of Natural Products of National Health and Family Planning Commission, Institute of Materia Medica, Peking Union Medical College and Chinese Academy of Medical Sciences, 1 Xian Nong Tan St., Beijing, 100050 China; 3 College of Life Science, Qufu Normal University, Qufu, 273165 Shandong China

**Keywords:** *Saccharomyces cerevisiae*, Glucosidase, Glucosyltransferase, Scutellarein 7-*O*-glucoside

## Abstract

**Background:**

Glycosylation of flavonoids is a promising approach to improve the pharmacokinetic properties and biological activities of flavonoids. Recently, many efforts such as enzymatic biocatalysis and the engineered *Escherichia coli* biotransformation have increased the production of flavonoid glucosides. However, the low yield of flavonoid glucosides can not meet the increasing demand for human medical and dietary needs. *Saccharomyces cerevisiae* is a generally regarded as safe (GRAS) organism that has several attractive characteristics as a metabolic engineering platform for the production of flavonoid glucosides. However, endogenous glucosidases of *S.cerevisiae* as a whole-cell biocatalyst reversibly hydrolyse the glucosidic bond and hinder the biosynthesis of the desired products. In this study, a model flavonoid, scutellarein, was used to exploit how to enhance the production of flavonoid glucosides in the engineered *S.cerevisiae*.

**Results:**

To produce flavonoid glucosides, three flavonoid glucosyltransferases (SbGTs) from *Scutellaria baicalensis* Georgi were successfully expressed in *E. coli,* and their biochemical characterizations were identified. In addition, to synthesize the flavonoid glucosides in whole-cell *S. cerevisiae*, SbGT34 was selected for constructing the engineering yeast. Three glucosidase genes (*EXG1*, *SPR1*, *YIR007W*) were knocked out using homologous integration, and the *EXG1* gene was determined to be the decisive gene of *S. cerevisiae* in the process of hydrolysing flavonoid glucosides. To further enhance the potential glycosylation activity of *S. cerevisiae*, two genes encoding phosphoglucomutase and UTP-glucose-1-phosphate uridylyltransferase involved in the synthetic system of uridine diphosphate glucose were over-expressed in *S. cerevisiae*. Consequently, approximately 4.8 g (1.2 g/L) of scutellarein 7-*O*-glucoside (S7G) was produced in 4 L of medium after 54 h of incubation in a 10-L fermenter while being supplied with ~3.5 g of scutellarein.

**Conclusions:**

The engineered yeast harbouring SbGT with a deletion of glucosidases produced more flavonoid glucosides than strains without a deletion of glucosidases. This platform without glucosidase activity could be used to modify a wide range of valued plant secondary metabolites and to explore of their biological functions using whole-cell *S. cerevisiae* as a biocatalyst.

**Electronic supplementary material:**

The online version of this article (doi:10.1186/s12934-016-0535-2) contains supplementary material, which is available to authorized users.

## Background

Flavonoids are a group of polyphenolic compounds that have various biological activities, such as antioxidant, anti-atherosclerosis and anti-tumour [1–3]. In plants, flavonoids typically exist in the form of glucosides [4], which is beneficial to enhance solubility and stability or to change the pharmacokinetic properties [5]. Generally, glycosylation, which is the final step in the biosynthesis of flavonoid glucosides, is catalysed by glycosyltransferases (GTs) which transfer sugar moieties from the activated donor molecules to specific acceptor molecules [6–8]. GTs are divided into 98 families (CAZy database, March 2016) according to their different donors and acceptors.

To date, with an increasing demand for flavonoid glucosides, natural plant extracts might not satisfy human medical and dietary needs, and chemical synthesis of glucosides requires many protection and deprotection steps, which result in non-sustainable and low yield [9]. Therefore, alternative approaches have been developed for the production of flavonoid glucosides, such as purified GTs in vitro [10] and the engineered *Escherichia coli* or *Saccharomyces cerevisiae* in vivo [11, 12]. So far, biocatalytic efforts to synthesize of flavonoid glucosides have largely focused on enzymatic synthesis and metabolic engineering of *E. coli.* The former is typically used to explore novel GTs and to characterize their new functions, and the latter automatically synthesizes sugar donors and directly catalyses the glycosylation of the target substrates supplied in the culture medium [10, 13, 14]. However, there are very few studies on the engineered yeast for the biosynthesis of flavonoid glucosides.

*Saccharomyces cerevisiae* is an attractive host organism for the production of flavonoid glucosides. Firstly, it is a unicellular eukaryote, which not only has the traits of prokaryotes, such as fast growth and advantages for culture and genetic manipulation, but also has the typical characteristics of eukaryotes, which allows for the modification of proteins after translation. Therefore, *S. cerevisiae* can be used to express other eukaryotic genes to synthesize the desired products. For example, it can functionally express *Arabidopsis thaliana* genes *UGD1* and *UXS3* to generate UDP-xylose [15]. In addition, *S. cerevisiae* is a generally regarded as safe (GRAS) organism that can be used in the production of pharmaceuticals and dietary supplements. However, whole-cell bioconversion of naringenin to naringenin 7-*O*-glucoside is impaired by the existence of endogenous glucosidases [16]. Additionally, Sabine Schmidt et al. identified several efficient glucosidases from yeast using an activity analysis of glucosidases in vitro and the corresponding commercial mutant strains [17]. Therefore, these glucosidases, which hydrolyse flavonoid glucosides, impede the use of *S. cerevisiae* as a host for the biotechnological production of flavonoid glucosides.

In the present work, an engineered *S. cerevisiae* strain was constructed to improve the production of flavonoid glucosides. Firstly, three glucosyltransferases (SbGTs) were obtained from *Scutellaria baicalensis* Georgi, and the SbGT34 exhibited the greatest activity toward scutellarein. Then, the glucosidase genes of *S. cerevisiae* were deleted to remove the hydrolysis of the glucoside products. To enhance the supplementation of the activated sugar donor UDP-glucose (UDP-Glu), two genes (encoding phosphoglucomutase 2 (*PGM2*) and UTP-glucose-1-phosphate uridylyltransferase 1 (*UGP1*)) involved in the biosynthesis of UDP-Glu were over-expressed in *S. cerevisiae*. Finally, an engineered yeast strain was constructed to enhance the production of flavonoid glucosides by combining the expression of *SbGT34*, *PGM2*, and *UGP1* with the deletion of glucosidases. Additionally, to facilitate the biosynthesis of flavonoid glucosides, assays on the glucose addition and pH were studied in the yeast strain. Bioconversion was further improved in a 10-L fermenter and approximately 1.2 g/L of scutellarein 7-*O*-glucoside was synthesized in 4 L of medium supplied with ~3.5 g of scutellarein.

## Results

### Biochemical characterization of recombinant SbGTs protein in vitro

**Fig. 1:**
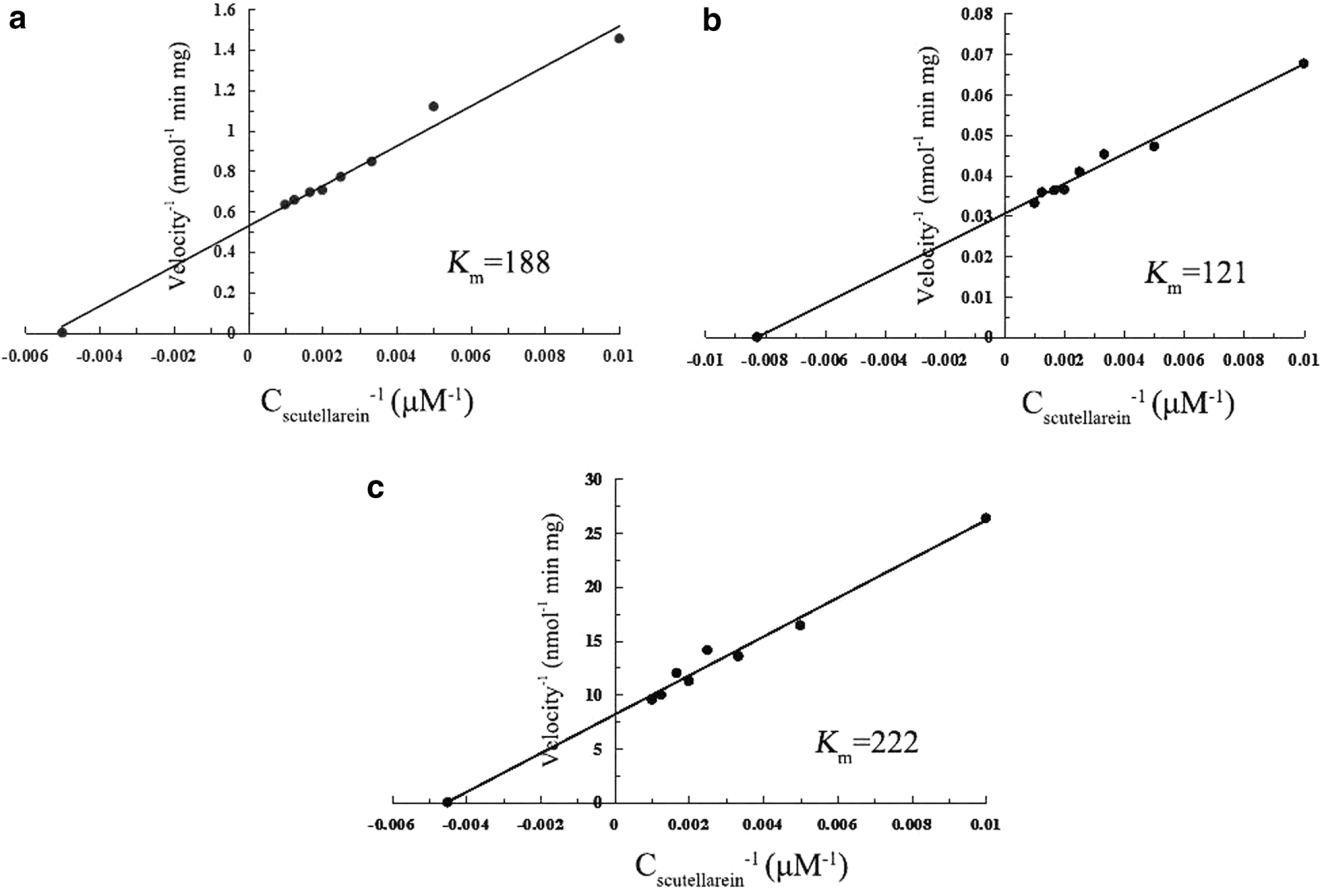
Determination of the kinetic parameters for SbGTs. The apparent *K*_m_ values were detected using scutellarein as an acceptor and UDP-Glu as a donor at 30 °C and a pH of 7.5. The apparent *K*_m_ values of SbGT30 (**a**), SbGT34 (**b**) and SbGT56 (**c**) were 188, 121 and 222 μM, respectively

Three SbGT genes [*SbGT30*, *SbGT34* and *SbGT56* (GenBank No. KU712253, KU712254 and KU712255)] encoding flavonoid glucosyltransferase with strictly regioselectivity of 7-hydroxyl group were identified and heterologously expressed in *E. coli*. The sugar donor specificity of these recombinant proteins was analyzed using scutellarein as a sugar acceptor and UDP-Glu, UDP-glucuronic acid (UDP-GA) and UDP-galactose (UDP-Gal) as sugar donors. The results showed that each enzyme only selectively accepted UDP-Glu as a sugar donor. These three SbGTs catalysed glucosyl transfer to scutellarein and produced a single glycosylated product, which was clearly identified as scutellarein 7-*O*-glucoside based on the comparison of its LC spectrum, MS and MS/MS fragments with authentic sample and the NMR spectra (Additional file 1: Supplemental results). The relative activity of each enzyme with the same amount of enzyme, scutellarein (0.2 mM) and UDP-Glu (0.6 mM) was the following: SbGT30, 76.3 %; SbGT34, 100 % and SbGT56, 24.6 %. The optimal pH and temperature for the SbGT34-mediated transfer of the glucosyl moiety to scutellarein were 7.5 and 30 °C, respectively (Additional file 1: Figure S2). A kinetic analysis of the SbGTs revealed that the *K*_m_ value of SbGT30 and SbGT56 for scutellarein were 155 and 183 %, respectively, of the *K*_m_ value of SbGT34 for scutellarein (Fig. 1). In addition, SbGT34 also catalyzed the glycosylation of other flavonoids (data not shown). Therefore, SbGT34 was selected to further catalyze the glucosidation of scutellarein in the engineered yeast.

### Deletion of *S. cerevisiae* glucosidase genes and its effects on the production of scutellarein 7-*O*-glucoside

In *S. cerevisiae*, the activity of glucosidases, which hydrolyse flavonoid glucosides, hinders the use of yeast as a host for the biotechnological production of glucosides [16, 17]. A flavonoid glucosyltransferase that glucosylated naringenin to naringenin 7-*O*-glucoside in vitro from *Dianthus caryophyllus*, which was expressed in *S. cerevisiae,* demonstrated the existence of endogenous glucosidase activity during whole-cell biocatalysis and was responsible for a greatly diminished product yield [16, 17]. In this study, we further confirmed the hydrolytic activity of the whole-cell yeast using luteolin 7-*O*-glucoside as a substrate (Additional file 1: Figure S3). The results showed that the yeast did hydrolyze β-glucoside, which was in agreement with previous studies [16, 17]. Therefore, if engineered *S. cerevisiae* was used to produce flavonoid glucosides, a key step in the construction of the engineered yeast is the deletion of the glucosidase genes of host strain. Thus, in this study, site-directed integration plasmids were transformed to the W303-1b strain to disrupt the glucosidase open reading frames. The resulting strains were generated through the targeted DNA integration mediated by homologous recombination. The knockout workflow is schematically shown in Additional file 1: Figure S4.

**Fig. 2:**
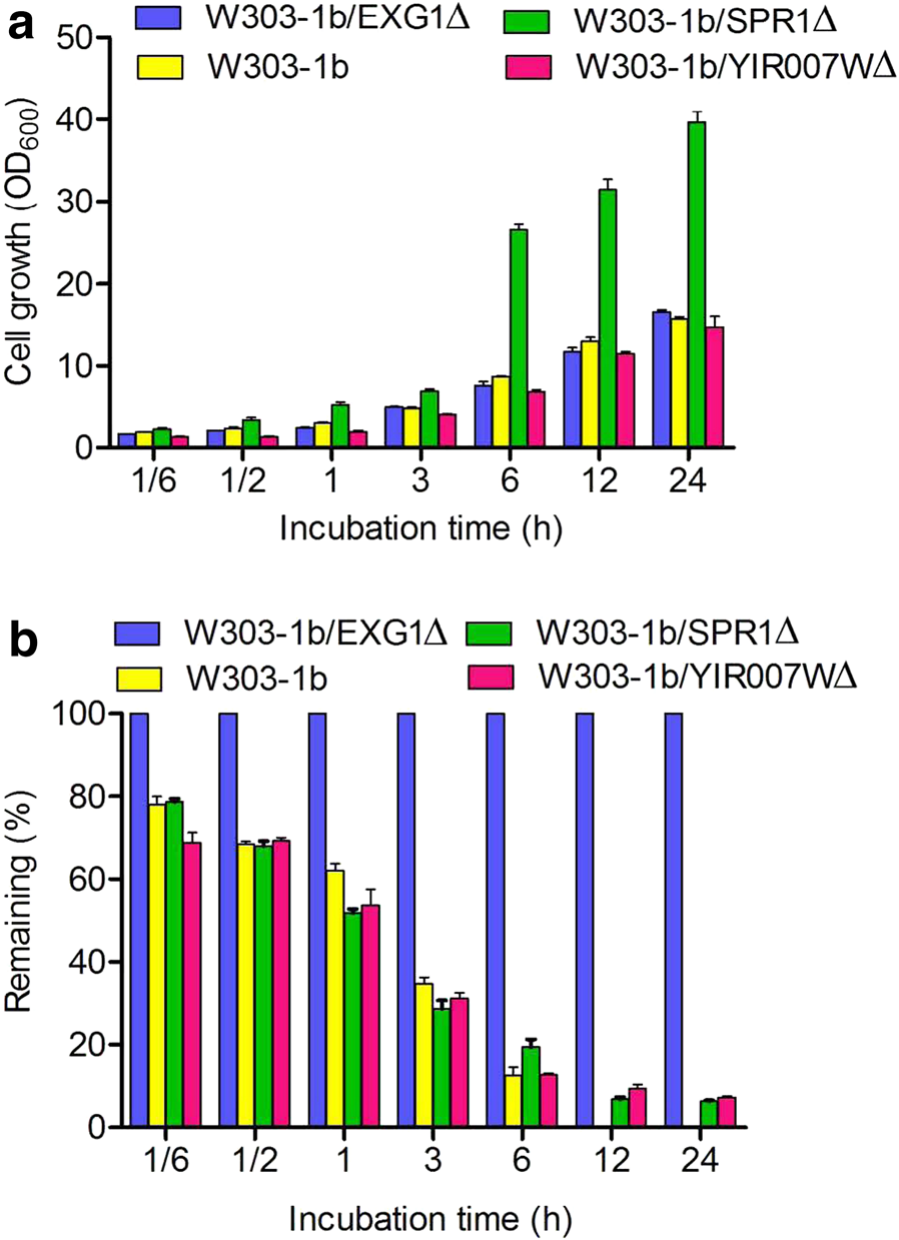
Hydrolytic activity to luteolin 7-*O*-glucoside in strains with the deletion of glucosidases. **a** Biomass (OD_600_). **b** The remaining rate of luteolin 7-*O*-glucoside in the liquid medium. The values are presented as the means, and the *error bars* show the SD (n = 3)

The integration of plasmids containing the selective markers *TRP1, ADE2* and *URA3,* used for the disruption of the *EXG1*, *SPR1* and *YIR007W* genes, respectively, of the W303-1b strain resulted in strains being selected on auxotrophic solid plates. Furthermore, positively integrated yeast strains were verified using DNA sequencing of the PCR-amplified fragments corresponding to the glucosidase locus from the genomic DNAs of the tested transformants. The growth rate of the strains with a deletion of the *EXG1* or *YIR007*W gene was similar to the wild-type strain, whereas the strain with the deletion of *SPR1* gene grew faster than the wild type strain (Fig. 2a).

To identify the glucosidase activity of the knockout strains, a degradation assay of flavonoid glucosides with whole-cell biocatalysis was performed using luteolin 7-*O*-glucoside as a substrate. When the glucoside was added to the culture medium with each knockout strain and the wild-type strain W303-1b, the remaining rate of the substrate in each catalyzing system was varied. The degradation of luteolin 7-*O*-glucoside to luteolin is presented in Fig. 2b. The luteolin 7-*O*-glucoside was dramatically diminished within 6 h in the wild-type strain W303-1b and could not be detected at 24 h. The strain W303-1b/EXG1∆ hardly hydrolyzed glucosides within 24 h, whereas the strains W303-1b/SPR1∆ and W303-1b/YIR007W∆ still showed high levels of hydrolytic activity. It is worthwhile to note that strain W303-1b/EXG1∆ did not hydrolyze glucosides even through it was incubated for 96 h during the whole-cell biocatalyst assay (data not shown). From these, the deletion of glucosidases was determined to be necessary for the glucosylation of whole-cell *S. cerevisiae* [16, 18].

**Fig. 3:**
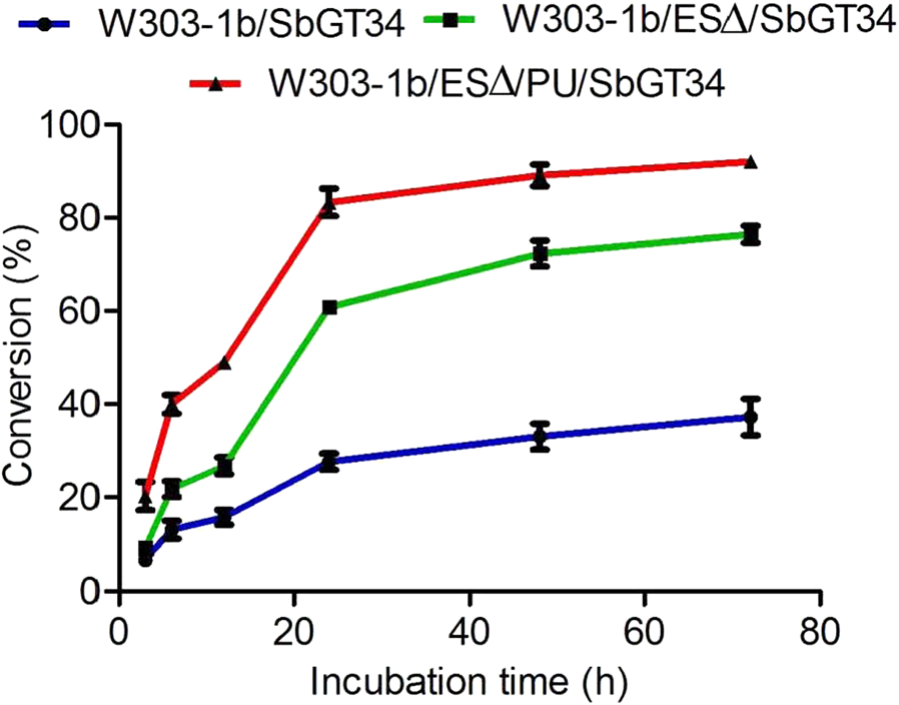
Differences in the level of scutellarein 7-*O*-glucoside produced by strains W303-1b/SbGT34, W303-1b/ES∆/SbGT34 and W303-1b/ES∆/PU/SbGT34 over time. Strains were incubated with 0.2 mM scutellarein. The values are presented as the means, and the *error bars* show the SD (n = 3)

To study strains harbouring SbGT34 to distinguish the biotransformation levels of substrates from flavonoids to flavonoid glucosides, 0.2 mM scutellarein was fed into the biocatalytic reaction systems. The results showed that in strain W303-1b/SbGT34, the conversion rate of scutellarein into its glucoside was increased slowly within the first 20 h, and glucoside achieved the greatest accumulation at 72 h, whereas the production of glucoside in strain W303-1b/ES∆/SbGT34 (E and S referred as deletion of genes *EXG1* and *SPR1*, respectively) was as approximately twice the production in strain W303-1b/SbGT34 at 72 h (Fig. 3) (i.e., the titres of product ranged from 33.4 to 68.6 mg/L). Therefore, according to the combination advantages of strain W303-1b/EXG1∆ that has almost no glucosidase activity, and strain W303-1b/SPR1∆ that grows fast, a double knockout strain W303-1b/ES∆, which had a growth rate similar to strain W303-1b/SPR1∆, was used for the further production of flavonoid glucosides.

### Bioconversion of scutellarein in vivo

**Fig. 4:**
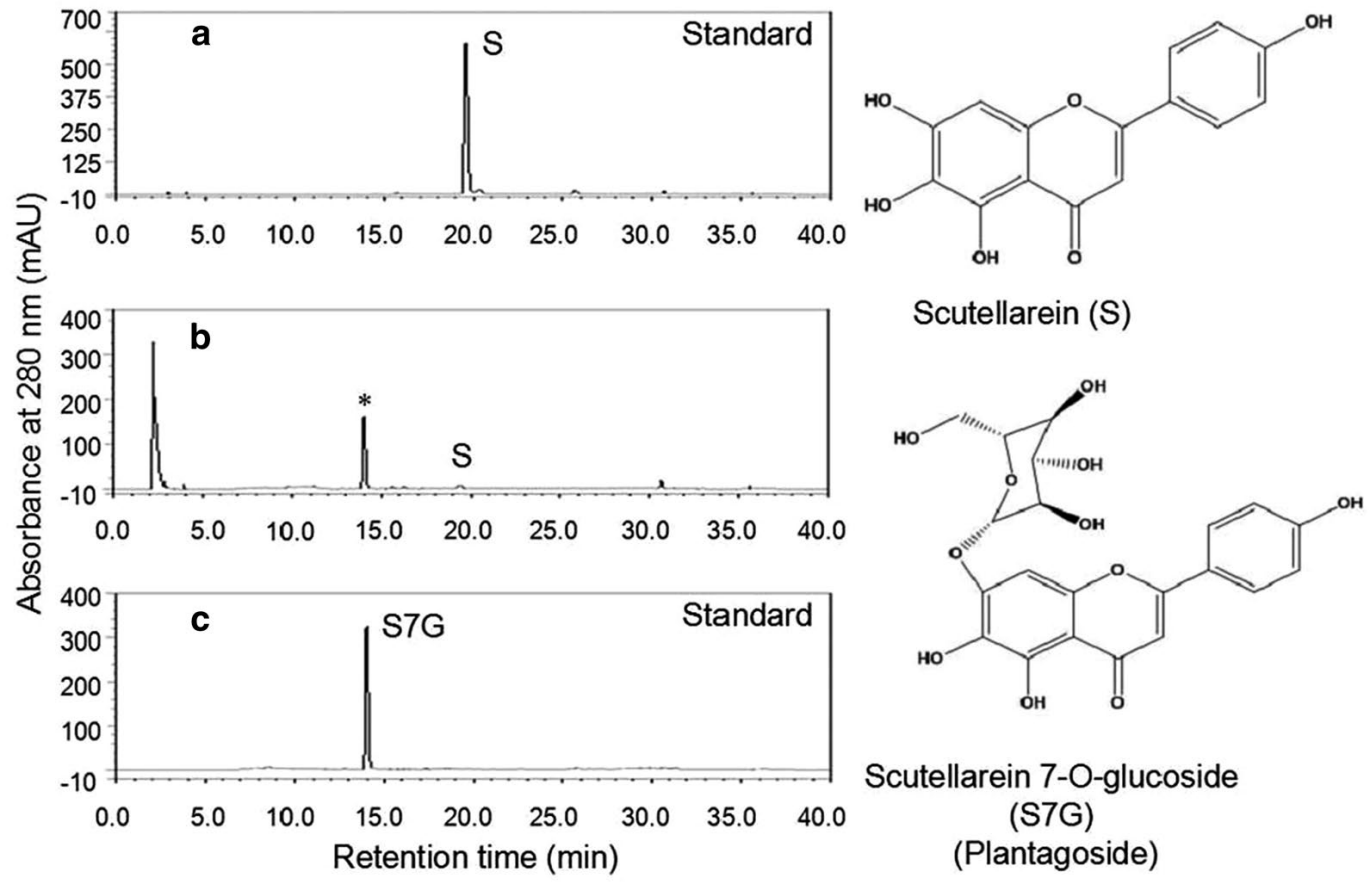
HPLC analysis for scutellarein 7-O-glucoside from the whole-cell biocatalyst assay using strain W303-1b/ES∆/PU/SbGT34. **a** HPLC profile for standard scutellarein. **b** HPLC profile for the biocatalysis system. **c** HPLC profile for standard scutellarein 7-*O*-glucoside

UDP-Glu is a critical endogenous donor in the biosynthesis of flavonoid glucosides in whole-cell *S. cerevisiae*. The biosynthetic pathway of UDP-Glu mainly includes two key enzymes: PGM2, which synthesizes glucose-1-phosphate from glucose-6-phosphate, and UGP1, which converts glucose-1-phosphate and UTP into UDP-Glu. The whole-cell *S. cerevisiae* produces endogenous UDP-Glu in the cytoplasm that could be used for the production of flavonoid glucosides [19–21], but does not comprise a GT for the glycosylation of flavonoids. To enhance the production of flavonoid glucosides in strain W303-1b/ES∆/PU/SbGT34 (P and U referred to the over-expression of genes *PGM2* and *UGP1*, respectively), SbGT34 was over-expressed under the control of the constitutive promoter along with the up-regulation of genes *PGM2* and *UGP1*. The results showed that strain W303-1b/ES∆/PU/SbGT34 was more effective (~92 % conversion rate) than strain W303-1b/ES∆/SbGT34 (~75 % conversion rate) (Fig. 3) (i.e., the titres of product ranged from 68.6 mg/L to 82.5 mg/L). The analysis of the reaction product from the HPLC profiles showed a product peak at a retention time of 14.1 min for scutellarein 7-*O*-glucoside compared to the standard scutellarein that was detected at 19.6 min at a UV absorbance of 280 nm (Fig. 4). The HPLC–MS/MS spectra showed an ion peak of scutellarein 7-*O*-glucoside at m/z = 471 [M + Na]^+^ (Additional file 1: Supplemental results). The negative experiment was carried out under identical conditions by supplementing scutellarein in the strain harbouring the empty vector, and no bioconversion of scutellarein was observed by analysing the HPLC results.

**Fig. 5:**
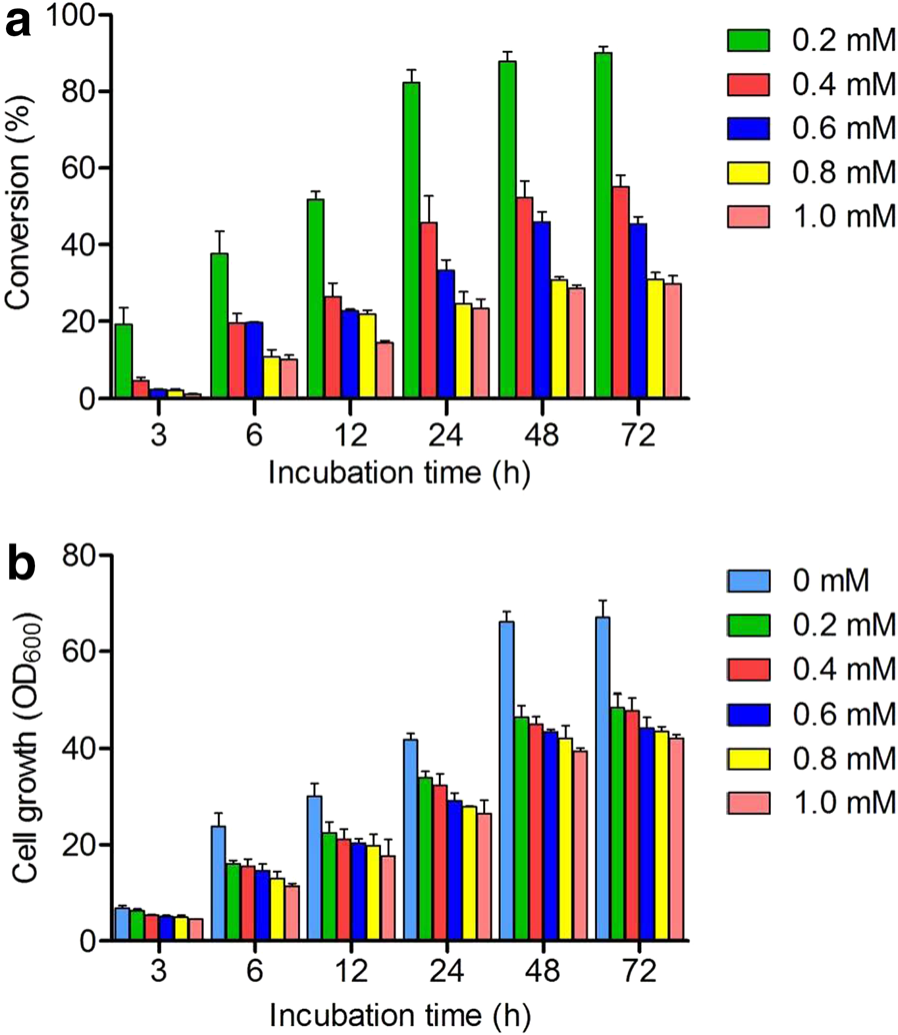
The influence of different concentrations of scutellarein on the cell growth and the conversion rate of scutellarein to scutellarein 7-*O*-glucoside. The concentrations of scutellarein were 0.2, 0.4, 0.6, 0.8 and 1.0 mM. **a** The conversion rate of scutellarein at different concentrations over time. **b** Biomass (OD_600_). The values are presented as the means, and the *error bars* show the SD (n = 3)

To explore the substrate inhibition on biotransformation and cell growth, different concentrations (0.2, 0.4, 0.6, 0.8, 1.0 mM) of scutellarein were fed into the biocatalytic reaction systems (strain W303-1b/ES∆/PU/SbGT34). The results showed that the bioconversion of scutellarein at each concentration gradient into its glucoside was rapidly increased in the first 48 h and then became static for 72 h, and the inhibition of cell growth was elevated with an increase in the concentration of the substrate (Fig. 5).

### Optimization of the production of flavonoid glucosides by regulating the concentration of glucose and the pH conditions in the medium

**Fig. 6:**
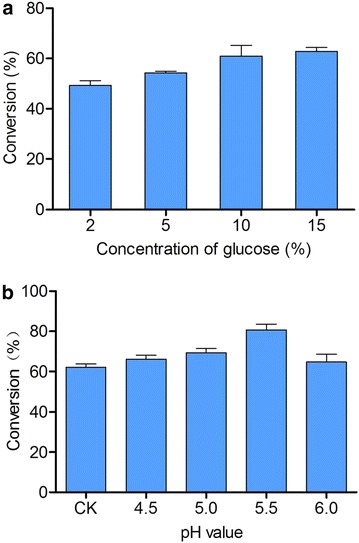
The influence of different concentrations of glucose and pH conditions on the fermentation. **a** A comparison of the conversion rate using 0.6 mM scutellarein in four different concentrations of glucose. **b** A comparison of the conversion rate using 0.6 mM scutellarein in four different pH conditions. CK represents the conversion rate in the unregulated medium, and the glucose concentration was 10 %. The values are presented as the means, and the *error bars* show the SD (n = 3)

During the fermentation process, glucose as the sole carbon source and as the precursor of the sugar donor together with the pH of medium is important for product synthesis. To further explore the potential of the recombinant strain W303-1b/ES∆/PU/SbGT34 for the production of scutellarein 7-*O*-glucoside, four different concentrations (2, 5, 10, 15 %) of glucose were supplemented in the synthetic complete (SC) medium with 0.6 mM scutellarein as the substrate. As shown in Fig. 6a, the conversion rate of scutellarein into its glucoside increased with the addition of glucose, and a similar transformation rate was observed at glucose concentrations of 10 and 15 % (i.e., the titres of product with the two concentrations of glucose was 161.4 and 168.9 mg/L, respectively). The supplementation of 10 % glucose concentration and 0.6 mM scutellarein acceptor were selected to further optimize pH.

Four different pH gradients (4.5, 5.0, 5.5 and 6.0) were designed for the biotransformation assay. The concentration of the phosphate buffer solution was 50 mM, and the conversion rate of the substrate was improved by approximately 20 % at pH 5.5 compared with the basic SC medium (Fig. 6b).

### Scale up for the production of scutellarein 7-*O*-glucoside using a fermenter

To verify the application of the engineered strain W303-1b/ES∆/PU/SbGT34 for the large-scale production of scutellarein 7-*O*-glucoside, optimized concentrations of scutellarein (1.0 mM at 0 h and 2.0 mM at 12 h) were added to the 4-L fermentation system in a 10-L fermenter. The temperature of fermenter was held at 30 °C and the pH was maintained at 5.5 by dripping of ammonia water during the entire process, and the concentration of glucose in the culture was 10 %. The culture medium was collected at a given time interval of 6 h and was analyzed using HPLC to monitor the conversion of substrate to its glucoside.

**Fig. 7:**
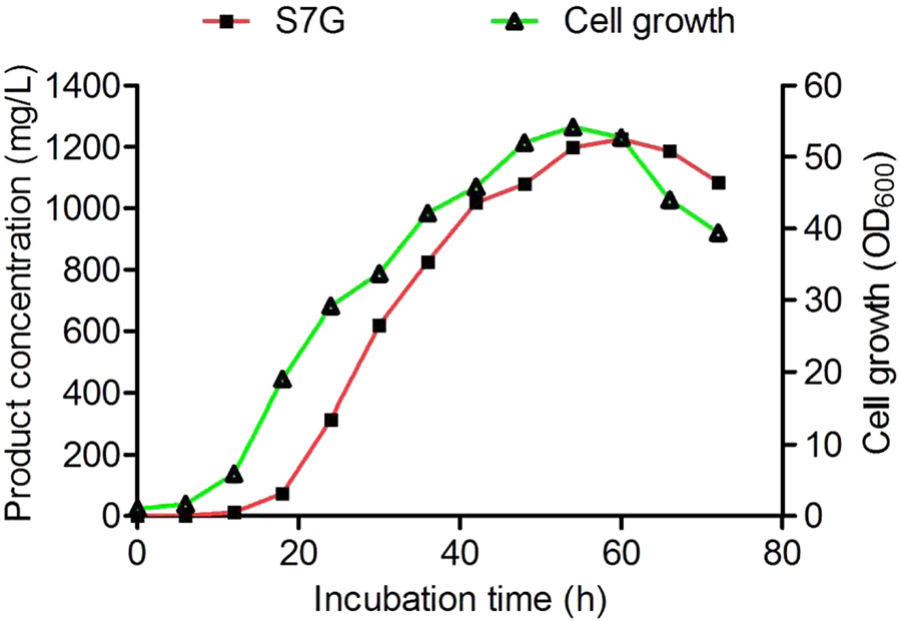
The scaled-up production of scutellarein 7-*O*-glucoside in the 10-L fermenter. Large-scale bioconversion in the fermenter using the SC medium supplemented with 10 % glucose. Scutellarein at concentrations of 1 and 2 mM were supplied in the reaction system at 0 and 12 h

After 12 h of fermentation, the supplied scutellarein was quickly begun to convert to scutellarein 7-*O*-glucoside; thus, additional 2.0 mM of scutellarein was added to the reaction system. Additionally, the fermentation condition was identical to the initial 12 h. Approximate 90 % of the supplemented substrate was converted into flavonoid glucoside after 54 h of fermentation. The overall calculations revealed that the production of scutellarein 7-*O*-glucoside was ~1.2 g/L (i.e., 2.7 mM or 4.8 g/4 L) from 3.0 mM of supplemented scutellarein (Fig. 7).

## Discussion

Dietary flavonoids and especially their glucosides, have attracted increasing attention in the past several decades for their considerable biological activities [4]. The glycosylation of flavonoids using GTs as the biocatalyst is of great general interest due to the synthesis of the desired compounds with high stereo- and regio-selectivity under mild conditions [4]. In recent studies, the exploration of GTs mainly focused on enzymology and the semi-synthesis of glucosides using engineered *E. coli* [4, 13, 22]. The in vitro recombinant enzyme provided a good model to study the enzymatic characteristics, and the in vivo biosynthesis of flavonoid glucosides using endogenous sugar donors could reduce the expense in engineered whole-cell *E. coli* system. Engineered whole-cell *S. cerevisiae* could also synthesize endogenous sugar donors and produce heterogeneous proteins. These virtues motivated us to investigate the glycosylation of engineered yeast in vivo.

To data, all most of GTs (e.g. UDP-glucose: anthocyanin 3-O-glucosyltransferase) reported are involved in the biosynthesis of anthocyanin, however, only a few studies have reported the isolation and characterization of GTs with strictly regio-selective glycosylation of flavonoid 7-hydroxyl group [23–29]. In this study, three additional flavonoid 7-*O*-glucosyltransferase genes from *S. baicalensis* were cloned and successfully expressed in *E. coli*. These three genes had high identities with the genes of the reported GTs from *Scutellaria* Species (Labiatae) (GenBank: AB031274, GU339042) [14, 24]. The results of the biochemical studies of SbGTs showed that the three SbGTs could regio-selectively catalyze scutellarein to scutellarein 7-*O*-glucoside, and only UDP-Glu could be used as a sugar donor. Additionally, SbGT34 had the greatest affinity towards scutellarein (Fig. 1). The amino acid sequence of the three SbGTs shared very high identities (more than 90 %) to the reported UBGT and SbUGT [14, 24], but SbGT34 showed the greatest catalytic efficiency for scutellarein compared with that of the SbGT56, which is most likely the counterpart of UBGT. Based on the alignment of their amino acid sequences and the homology modeling (data not shown), a significant deletion of seven amino acid residues in SbGT30 and SbGT34 was within the calculated loop region between the N- and C-terminal domains with similar Rossmann-like GT-B folds among the plant flavonoid GTs (Additional file 1: Figure S5). Additionally, there are several different amino acid residues between SbGT30 and SbGT34 highlighted (Additional file 1: Figure S5), of which the function of these amino acid residues need to be further tested in detail.

The biocatalytic production of flavonoid glucosides using yeast as a biocatalyst is impaired by the metabolic activity of yeast glucosidases, which hydrolyze the glucosidic bond between aglycone and glucosyl. A GT cloned from *D. caryophyllus* that could glucosylate naringenin to naringenin 7-*O*-glucoside was expressed in whole-cell *S. cerevisiae.* However, the endogenous glucosidases reversely hydrolyzed naringenin 7-*O*-glucoside during whole-cell biocatalysis, resulting in a low final glucoside titre [16]. Sabine Schmidt et al. had identified the three yeast β-glucosidases in yeast: EXG1, SPR1 and YIR007W, which played significant roles in the hydrolysis of flavonoid glucosides [17]. In this work, these three glucosidase genes were disrupted in the engineered yeast. The *EXG1* gene was identified in vivo as the decisive gene for the degradation of flavonoid glucosides. Strains with the deletion of the *EXG1* gene did not hydrolyze the flavonoid glucosides (Fig. 2b), and the growth rate of the strain with the deletion of the *SPR1* gene was greater than the wild strain and other recombinant strains (Fig. 2a). The *SPR1* gene encodes a sporulation-specific glucanase and is transcribed only during the late stages of sporulation [30]. Deletion of the *SPR1* gene contributes to change the proceeding of sporulation and promotes cell growth. The high growth rate of the strain with the deletion *SPR1* gene could shorten the process of fermentation. The conversion rate of flavonoid in strain W303-1b/ES∆/SbGT34 increased twofold compared with the rate in strain W303-1b/SbGT34 (Fig. 3). Therefore, the strain with the disruption of the *EXG1* and *SPR1* genes is proposed as an option to develop a platform for the expression of GTs and for the production of flavonoid glucosides in yeast transformants.

The combined strategy of deletion of glucosidases and the introduction of GTs plays pivotal roles in generating flavonoid glucosides. Plant GTs expressed in *E. coli* have been reported for the glycosylation of flavonoids [13, 22]. Based on the expression of the GTs in *E. coli*, the titre of the flavonoid glucosides was less than 100 mg/L in the culture broth [25, 26]. The two main factors for the biosynthesis of the desired products might be related to the availability of intracellular UDP-sugars and low activity of the GTs. Recently, a number of studies have primarily focused on enhancing endogenous UDP-sugars by over-expressing genes that are part of the biosynthetic pathway of UDP-sugars and on exploring efficient GTs [22, 31–33]. In this study, the successful deletion of glucosidases and the expression of the *S. baicalensis* flavonoid GT in the engineered yeast for the whole-cell biocatalysis facilitated the bioconversion of aglycones and significantly improved the production of scutellarein 7-*O*-glucoside. The scale-up feasibility of in vivo glycosylation has been applied for the large-scale production of scutellarein 7-*O*-glucoside by optimizing the appropriate fermentation conditions. Consequently, up to 1.2 g/L of scutellarein 7-*O*-glucoside was produced after 54 h of fermentation (Fig. 7). As the titre of the desired products was remarkably high, the engineered strain W303-1b/ES∆/PU/SbGT34 is the most efficient strain for the production of flavonoid glucosides in yeast ever reported.

## Conclusions

The combined strategy of the deletion of endogenous glucosidases and the introduction of heterogenous GTs along with the up-regulation of biosynthetic pathway of UDP-Glu presents higher efficiency of the production of flavonoid glucosides. Through replacing other GTs and the regulating of biosynthetic pathway of UDP-sugars, it is applicable to extend the regio-specific glycosylation to more secondary metabolites as pharmaceutical ingredients for future clinical application.

## Methods

### Strains

*Escherichia coli* DH5α (TransGen, Beijing, China) was used for propagation and manipulation of the recombinant DNA, and *E. coli* BL21 (DE3) was employed for expression of the recombinant enzymes. *S. cerevisiae* W303-1b (*MATα ade2*-*1 leu2*-*3, 112 his3*-*11, 15 ura3*-*1 trp1*-*1*) was used for construction of engineering strain as a whole-cell biocatalyst for production of flavonoid glucosides. *E. coli* strains were cultured at 37 °C in LB medium (5 g/L yeast extract, 10 g/L tryptone and 10 g/L NaCl), and yeast transformants were grown at 30 °C in YPD medium (10 g/L yeast extract, 20 g/L tryptone and 20 g/L glucose).

### Expression and purification of recombinant SbGTs

To prepare the recombinant SbGT enzymes, the verified cDNA clones corresponding to SbGT30, SbGT34 and SbGT56 were amplified, digested with restriction enzymes *Nco* I and *Bam*H I, and then ligated into the same sites of the expression vector pTWIN1B, a derivative of the plasmid pTWIN1 (NEB, Biolabs) (Additional file 1). After the verification of the sequences, the resulting pTWIN1B-SbGTs plasmids and a control pTWIN1B were transformed into *E. coli* BL21 (DE3) for heterologous expression. Luria–Bertani (LB) medium (10 mL) containing 100 μg/mL of ampicillin was inoculated with 1 mL of an overnight culture corresponding to each selected genes and a control vector. Cells were grown at 37 °C with shaking (200 rpm) until the OD_600_ achieved 0.4–0.6. The recombinant N-terminal CBD-tagged SbGTs were induced with 130 μg/ml isopropy β-D-thiogalactoside (IPTG) for 20 h at 16 °C with shaking (165 rpm). Cells were harvested by centrifugation at 8000 *g* for 5 min at 4 °C. Pellets were resuspended in 100 mL of chilled binding buffer (20 mM HEPES, 500 mM NaCl, NaOH solution was added until pH was 8.5). Cells were disrupted with 800 bar high pressure using high pressure homogenizer and the cell debris was removed by centrifugation at 10,000 *g* and 4 °C for 30 min.

The cleared supernatant was immediately applied to 10 mL of chitin resin (NEB, Biolabs) loaded in a column that was pre-equilibrated with binding buffer. The chitin resin was subsequently washed with 100 mL of the washing buffer (20 mM HEPES, 500 mM NaCl, pH 8.5). Then, the washing buffer was replaced by the cleavage buffer (20 mM HEPES, 50 mM NaCl, pH 6.5) and the recombinant protein was cleaved at 4 °C for 3 days. Elution was carried out with 25 mL of the cleavage buffer, and the recombinant protein was desalted in 10-times diluted cleavage buffer and was then lyophilized. Protein purity was confirmed by SDS-PAGE to be >90 % (Additional file 1: Figure S1), and the protein concentration for all studies was determined by the protein quantitative kit (Bradford) (Bio-Rad, USA).

### Glycosyltransferase activity assays in vitro

Three SbGT genes, *SbGT30*, *SbGT34* and *SbGT56*, were identified and heterologously expressed in *E. coli*. The donor specificity of these recombinant proteins was analyzed using scutellarein as a glucosyl acceptor and UDP-Glu, UDP-GA and UDP-Gal as sugar donors. The reaction mixture (200 μL) for the GTs contained 50 mM citrate buffer (pH 6.5), 0.6 mM UDP-Glu, 0.2 mM substrate dissolved in dimethylsulfoxide (DMSO) and 10 μg of the purified SbGTs. Activity assays, which were initiated through the addition of enzymes, were incubated at 30 °C for 30 min and were terminated by adding 200 mL of methanol. The reaction mixture was subjected to HPLC analysis for the detection of the reaction product.

### Effects of pH, temperature and divalent metal ions on enzyme activity

To test the necessity of divalent metal ions for SbGT34, CaCl_2_, CoCl_2_, CuCl_2_, MgCl_2_, FeCl_2_, MnCl_2_, ZnCl_2_ and EDTA were used individually at a final concentration of 5 mM. To study the optimal pH, the enzymatic reaction was performed in various reaction buffers with pH values in the range of 4.0–6.5 (citrate buffer), 6.0–8.0 (phosphate buffer) and 7.5–9.0 (Tris–HCl buffer). To assay for the optimal reaction temperature, the reaction mixtures were incubated at different temperatures (0–70 °C). The assays were performed with UDP-Glu as a donor and scutellarein as an acceptor.

### Determination of kinetic parameters

Assays were performed in a final volume of 200 μL consisting of 50 mM Tris–HCl (pH 7.5) at 30 °C, and contained constant concentrations of enzyme (10 μg) and saturating UDP-Glu (2 mM) while varying the scutellarein concentration (0.1–1.0 mM). Reactions were terminated at 10 min (where the rate of product formation was determined to be linear) by adding an equal volume of ice cold methanol. Mixtures were filtered and analyzed using reverse-phase HPLC, and the total per cent conversion was calculated as the per cent of the total peak area of the substrate and product. All experiments were performed in triplicate. The value of *K*_m_ was calculated using a Lineweaver–Burk plot.

### Construction of yeast expression plasmids and yeast transformation

All strains and plasmids used in this study are listed in Table 1. The plasmid construction and yeast transformation methods were same as in [34]. All plasmids were constructed using conventional restriction enzyme-mediated cloning methods. Based on the nucleotide sequences of the target genes, development of primer sets were designed and used to amplify gene fragments by PCR (Additional file 1: Tables S1–S6). The δ DNA-mediated integrative expression vector was used for the cloning of the polymerase chain reaction (PCR) products and the expression of the genes [35]. The acquired plasmids were linearized by digestion with the restriction enzyme *Not* I or *Hind* III and were transformed into the *S. cerevisiae* using the lithium acetate method. Transformants were selected using yeast extract peptone dextrose (YPD) agar plates containing the antibiotics Geneticin (G418, 4 mg/mL) or Hygromycin B (HygB, 1 mg/mL), and the double homologous recombination of the target genes was verified by PCR using the corresponding primers and Sanger sequencing using the isolated genomic DNA as a template. The repeated introduction of the marker genes was implemented via a loxP-marker-loxP gene disruption cassette [36].

**Table 1:** Strains and plasmids used in this study

Strains or plasmids	Relevant properties	Source or reference
Strains		
*E. coli* trans1-T1	F^−^ *φ80(lacZ)∆M15 ∆lacX74 hsdR(rK*− *mK*+*)∆recA1398 endA1 tonA*	Our lab
*E. coli* BL21 (DE3)	F^−^ *ompT hsdS* (*rBB*-*mB* ^−^) *gal dcm*	Our lab
*S. cerevisiae* W303-1b	*MATα ade2*-*1 leu2*-*3, 112 his3*-*11, 15 ura3*-*1 trp1*-*1*	Our lab
W303-1b/EXG1∆	W303-1b derivative with pEZ-EXG36-Trp1, EXG1∆::Trp1	This study
W303-1b/SPR1∆	W303-1b derivative with pEZ-SPR36-ADE2, SPR1∆::Ade2	This study
W303-1b/YIR007W∆	W303-1b derivative with pEZ-YIR36-Ura3, YIR007W∆::Ura3	This study
W303-1b/ES∆	W303-1b derivative with pEZ-EXG36-Trp1 and pEZ-SPR36-ADE2, EXG1∆::Trp1, SPR1∆::Ade2	This study
W303-1b/SbGT34	W303-1b derivative with pδGAPg-SbGT34	This study
W303-1b/ES∆/SbGT34	W303-1b/ES∆ derivative with pδGAPg-SbGT34	This study
W303-1b/ES∆/PU/SbGT34	W303-1b/ES∆ derivative with pδGAPg-PGM2, pδGAPh-UGP1, and pδGAPg-SbGT34	This study
Plasmids		
pTWIN1B	A derivative of pTWIN1, an inducible expression vector with ampicillin resistant.	Our lab
pTWIN1B-SbGT30	pTWIN1B derivative with the *SbGT30* gene DNA	This study
pTWIN1B-SbGT34	pTWIN1B derivative with the *SbGT34* gene DNA	This study
pTWIN1B-SbGT56	pTWIN1B derivative with the *SbGT56* gene DNA	This study
pEZ-EXG36-TRP1	Integration vector with Homologous fragment of *EXG1* and selective marker *TRP1*	This study
pEZ-SPR36-ADE2	Integration vector with Homologous fragment of *SPR1* and selective marker *ADE2*	This study
pEZ-YIR36-URA3	Integration vector with Homologous fragment of *YIR007W* and selective marker *URA3*	This study
pδGAPg	pBluescript II KS(+) derivative with homologous δ region, *P*_*gap*_, *T*_*pgk1*_, G418^r^	[34]
pδGAPh	pBluescript II KS(+) derivative with homologous δ region, *P*_*gap*_, *T*_*pgk1*_, HygB^r^	[34]
pδGAPg-PGM	pδGAPg derivative with *PGM2* from *S. cerevisiae*, G418^r^	This study
pδGAPh-UGP1	pδGAPh derivative with *UGP1* from *S. cerevisiae*, HygB^r^	This study
pδGAPg-SbGT34	pδGAPg derivative with *SbGT34* from *S. baicalensis*, G418^r^	This study

### Deletion of glucosidases in *S. cerevisiae*

In *S. cerevisiae*, glucosidases EXG1, SPR1 and YIR007W have previously been identified as the three yeast β-glucosidases previously [17]. To disrupt these glucosidase genes, three homologous integration vectors were constructed (Additional file 1: Methods). Then, 5 μg of the resulting plasmids that were linearized by the digestion with restriction enzyme *Not* I or *Hind* III were transformed into *S. cerevisiae* using the lithium acetate method. Transformants were identified on synthetic selective medium agar plates without tryptophan (for gene *EXG1*), adenine (for gene *SPR1*) or uracil (for gene *YIR007W*), respectively [37]. The double homologous recombination of the glycosidase locus was verified by PCR using the corresponding primers and the corresponding genomic DNA as a template. The amplified DNA fragments of the positive constructs were further verified by DNA sequencing (Taihe, Beijing, China).

### Analysis of the glucosidase activity in the knockout strains

After each successful deletion, strains W303-1b, W303-1b/EXG1∆, W303-1b/SPR1∆ and W303-1b/YIR007W∆ were grown at 30 °C in YPD medium for 24 h, and the culture was then inoculated into fresh SC medium (2 % w/v glucose, 0.67 % w/v yeast nitrogen base without amino acids and 2 g/L complete amino acid mixture) at 1 %, and the cells were grown at 30 °C for approximately 10 h. The initial OD_600_ of the yeast seeds in the SC media was adjusted to 1.0 and 0.2 mM luteolin 7-*O*-glucoside was then added to the medium. The 1 mL reaction mixture in 5-mL reaction flasks was incubated at 30 °C for 10, 30 min, 1, 3, 6, 12 and 24 h. After the completion of the reaction, the reaction products of three independent experiments were lyophilized and extracted using 500 μL of methanol three times. The combined extracts were evaporated and then re-dissolved in 1 mL of methanol. The hydrolytic rate of each strain for luteolin 7-*O*-glucoside was determined using HPLC analysis.

### Analysis of the dynamic effects of glucosidase activity on the production of the desired glucosides

To further confirm whether the strains harbouring SbGT34 with or without the deletion of glucosidase genes had different substrate conversion rates, strains W303-1b/SbGT34 and W303-1b/ES∆/SbGT34 were selected to explore the remaining glucosidase activity. Strains W303-1b/SbGT34 and W303-1b/ES∆/SbGT34 were grown at 30 °C in YPD medium for 24 h, and the culture was then inoculated into fresh SC medium at 1 % and the cells were grown at 30 °C for approximately 10 h. The initial OD_600_ of the yeast seed in the SC media was adjusted to 1.0 and 0.2 mM scutellarein was then added to the medium. The 1-mL reaction mixture was incubated at 30 °C for 3, 6, 12, 24, 48 and 72 h in 5-mL reaction flasks. After the completion of the reactions, the reaction products were processed as mentioned above. The conversion rate of each strain for scutellarein was determined using HPLC analysis.

### Assay on *S. cerevisiae* whole-cell biocatalyst

Integrative *S. cerevisiae* transformants were grown overnight at 30 °C in YPD medium until the OD_600_ was approximately 3.0 as a seed culture. One hundredth of the volume of the seed culture was inoculated into the SC medium and was grown at 30 °C for approximately 10 h, and the OD_600_ was adjusted to 1.0 using fresh SC medium. Substrates were added to 1 mL of the regulated cell suspension. All of the results shown in this paper were obtained from at least three independent experiments.

Yeast strain W303-1b/ES∆/PU/SbGT34 at the same cell density (initial OD_600_ 1.0) was used for the substrate inhibition tests. Different concentrations of scutellarein (0.2, 0.4, 0.6, 0.8, 1.0 mM) dissolved in DMSO were used to biotransform in a 1-mL culture volume in 5-mL reaction flasks. Three independent experiments were removed from the shaker for each concentration and for each reaction time interval. The reaction solutions were lyophilized, and the samples were then extracted using 500 μL methanol three times. The three methanol fractions were merged and completely volatilized, and then re-suspended into 1 mL of methanol, and subjected to HPLC analysis. The final conversion of substrate was calculated according to a standard curve of scutellarein and scutellarein 7-*O*-glucoside.

### Glucose supplementation and pH optimization

The yeast strain W303-1b/ES∆/PU/SbGT34 was used for glucose supplementation and pH optimization in a biotransformation reaction. Firstly, four different concentrations of glucose (2, 5, 10, 15 %) were supplemented in the SC medium with 0.6 mM scutellarein as a substrate to determine the optimum concentration of glucose for biotransformation. Secondly, when the optimum concentration of glucose was determined, 50 mM phosphate buffer solution was added to the fermentation medium in four pH gradients (4.5, 5.0, 5.5, 6.0) to ascertain the optimal pH for bioconversion. The original cell density and disposing methods of the samples were same as mentioned above. Finally, HPLC analysis was carried out, and the conversion rate of the substrate into product was determined.

### Whole-cell biocatalyst assay in the fermenter system

A 10-L glass autoclavable fermenter system (Beauty, Shanghai, China) was used for the large-scale analysis of the strains harbouring SbGT34 as a whole-cell biocatalyst. The strain W303-1b/ES∆/PU/SbGT34 that was cultured overnight (200 mL) was transferred into the fermenter system containing 4 L of SC medium, and the original OD_600_ was adjusted to 1.0. The culture was fed with 1.0 mM scutellarein (dissolved in DMSO) (at 0 h) and 2.0 mM (at 12 h) as a substrate, and the incubation temperature and dissolved O_2_ were 30 °C and 25 %, respectively. The pH of the medium was regulated to remain at approximately 5.5 through the addition of a 12.5 % ammonia solution. Samples were harvested at 6 h intervals, and the growth of the cells was measured at 600 nm. The resulting solution fractions (culture medium) were disposed as mentioned above and analysed using HPLC.

### Product analysis and quantification

The culture extracts dissolved in methanol were directly performed on reverse-phase HPLC connected to a C18 column (Mightysil RP-18 GP 4.6 × 250 mm, 5 μm) at 280 nm using binary conditions of H_2_O (0.05 % trifluoroacetic acid, mobile phase A) and 100 % acetonitrile (ACN) (mobile phase B) at a flow rate of 1 ml/min for 40 min. The analyses of the substrates and their products were conducted using the following gradient program: 0–25 min (10–35 % B), 25–27 min (35–100 % B), 27–32 min (100 % B), 32–35 min (100–10 % B), 35–40 min (10 % B). To quantify the flavonoid glucosides, a calibration curve of scutellarein 7-*O*-glucoside was created using 0.05, 0.1, 0.2, 0.5, 0.6, 0.8, 2.0 and 4.0 mM concentrations.

## Additional file

10.1186/s12934-016-0535-2 **Table S1.** Primers used for cloning of *S. baicalensis* SbGT cDNAs. **Table S2.** Primers used for cloning of *S. cerevisiae* EXG1 (GenBank No. M34341) homologous DNA fragments. **Table S3.** Primers used for cloning of *S. cerevisiae* SPR1 (GenBank No. NM_001183609) homologous DNA fragments. **Table S4.** Primers used for cloning of *S. cerevisiae* YIR007W (GenBank No. NM_001179529) homologous DNA fragments. **Table S5.** Primers used for cloning of *S. cerevisiae* PGM2 (GenBank No. NM_001182605) gene. **Table S6.** Primers used for cloning of *S. cerevisiae* UGP1 (GenBank No. NM_001179601) gene. **Figure S1.** SDS-PAGE analysis of recombinant SbGTs purified by affinity chromatography. **Figure S2.** Effects of various divalent metal ions, pH, temperature on enzyme activity of SbGT34. **Figure S3.** Activity analysis of whole-cell glycosidase in wild-type *S. cerevisiae* W303-1b using luteolin 7-*O*-glucoside as a substrate. **Figure S4.** Diagrammatic sketch of the knockout of glucosidase genes. **Figure S5.** A multiple alignment of the amino acid sequences of SbGT30, SbGT34, SbGT56, UBGT, SbUGT and UGT71G1. **Figure S6.** Schematic diagram of biosynthetic pathway of scutellarein 7-*O*-glucoside.

## Abbreviations

UDP: uridine diphosphate
UTP: uridine triphosphate
GT: glucosyltransferase
UDP-Glu: UDP-glucose
UDP-GA: UDP-glucuronic acid
UDP-Gal: UDP-galactose
PGM2: phosphoglucomutase 2
UGP1: UTP-glucose-1-phosphate uridylyltransferase 1
