# Supplementary material for: Engineering Saccharomyces cerevisiae with the deletion of endogenous glucosidases for the production of flavonoid glucosides
Source: Microb Cell Fact. 2016 Aug 4;15:134. doi: 10.1186/s12934-016-0535-2 (PMC4973555; doi:10.1186/s12934-016-0535-2)
Supplement: Supplementary file 1 — 10.1186/s12934-016-0535-2 Table S1. Primers used for cloning of S. baicalensis SbGT cDNAs. Table S2. Primers used for cloning of S. cerevisiae EXG1 (GenBank No. M34341) homologous DNA fragments. Table S3. Primers used for cloning of S. cerevisiae SPR1 (GenBank No. NM_001183609) homologous DNA fragments. Table S4. Primers used for cloning of S. cerevisiae YIR007W (GenBank No. NM_001179529) homologous DNA fragments. Table S5. Primers used for cloning of S. cerevisiae PGM2 (GenBank No. NM_001182605) gene. Table S6. Primers used for cloning of S. cerevisiae UGP1 (GenBank No. NM_001179601) gene. Figure S1. SDS-PAGE analysis of recombinant SbGTs purified by affinity chromatography. Figure S2. Effects of various divalent metal ions, pH, temperature on enzyme activity of SbGT34. Figure S3. Activity analysis of whole-cell glycosidase in wild-type S. cerevisiae W303-1b using luteolin 7-O-glucoside as a substrate. Figure S4. Diagrammatic sketch of the knockout of glucosidase genes. Figure S5. A multiple alignment of the amino acid sequences of SbGT30, SbGT34, SbGT56, UBGT, SbUGT and UGT71G1. Figure S6. Schematic diagram of biosynthetic pathway of scutellarein 7-O-glucoside. [file 12934_2016_535_MOESM1_ESM.doc]

**ADDITIONAL FILE 1**

**List of Tables**

Table S1. Primers used for cloning of *S. baicalensis* SbGT cDNAs.

Table S2. Primers used for cloning of *S. cerevisiae EXG1* (GenBank NO. M34341) homologous DNA fragments.

Table S3. Primers used for cloning of *S. cerevisiae SPR1* (GenBank NO. NM_001183609) homologous DNA fragments.

Table S4. Primers used for cloning of *S. cerevisiae YIR007W* (GenBank NO. NM_001179529) homologous DNA fragments.

Table S5. Primers used for cloning of *S. cerevisiae* *PGM2* (GenBank NO. NM_001182605) gene.

Table S6. Primers used for cloning of *S. cerevisiae* *UGP1* (GenBank NO. NM_001179601) gene.

**List of figures**

Figure S1. SDS-PAGE analysis of recombinant SbGTs purified by affinity chromatography.

Figure S2. Effects of various divalent metal ions, pH, temperature on enzyme activity of SbGT34.

Figure S3. Activity analysis of whole-cell glycosidase in wild-type *S. cerevisiae* W303-1b using luteolin 7-*O*-glucoside as a substrate.

Figure S4. Diagrammatic sketch of the knockout of glucosidase genes.

Figure S5. A multiple alignment of the amino acid sequences of SbGT30, SbGT34, SbGT56, UBGT, SbUGT, and UGT71G1.

Figure S6. Schematic diagram of biosynthetic pathway of scutellarein 7-*O*-glucoside.

**Supplemental results**: identification of glucosylation products. MS, ^1^H NMR, ^13^C NMR data of scutellarein 7-*O*-glycoside.

**Table S1. Primers used for cloning of *S. baicalensis* SbGT** cDNAs.

| Primers | Oligonucleotide sequences (5'-3') |
| --- | --- |
| UDPG1 | GATTCCATTATAGAATCTTGAAATG |
| UDPG2 | ATCTTGACCATATGGGACAACTCCACATAGTCC |
| UDPG3 | CAAGAACCCTCGAGAGTTTAAGCCCTGTTTCATAGGAGG |
| UDPG4 | CGTAATTTATATGAGACAAGAACC |
| UDPG5 | TCATGTGTGGCACCAAATCCACA |
| UDPG_E1 | GGATCCGGAATTCCACCATCACCATCACCATGGACAACTCCACATAGTCCTT |
| UDPG_E2 | CAAGAACCCTCGAGTTAGTTTAAGCCCTGTTTCATAGGA |
| Twin1-B1 | GATATACCATGGGCAGCAGCCATCAT |
| Twin1-B2 | TCAGTAAGATCTTTAGCAGCCGGATCTCAGTG |
| SBUGT_N1 | AGATATACATATGGGACAACTCCACATAGTCCTTG |
| SBUGT_X1 | GAGCTCTCTAGACTAGTTTAAGCCCTGTTTCATAGGAGG |

**Table S2. Primers used for cloning of *S. cerevisiae EXG1*** (GenBank NO. M34341) homologous DNA fragments.

| Primers | Oligonucleotide sequences (5'-3') |
| --- | --- |
| EXG_1 | TACTCGCTACCCCAGTCCCTGCA |
| EXG_2 | GTTCGAGGCTTTCCGTACAAATG |
| EXG_3 | GAATTCGGTACCGCGGCCGCTCGACGAATATCACTTCTGT |
| EXG_4 | GATCATCATCGCTAGCCGATGATGATGCATGCCGATACCAATAACAGTGTCC |
| EXG_5 | CATCGGCTAGCGATGATGATCGTTAACGTACCGGAGTTTTGAATGAATCC |
| EXG_6 | TACATGGAGCTCGCGGCCGCGAGGGATCTCTGTAAAATGTTGGAT |
| EXG_7 | AAGTGTACCATATAAAATAATGTC |
| EXG_8 | ATCGTATATTCTATAAGTGTACC |
| Trp_EXG1 | AGAGAGGCTAGCGCGCGTTTCGGTGATGACG |
| Trp_EXG2 | CTGTGCGTTAACTATTTCACACCGCATAGATC |

**Table S3. Primers used for cloning of *S. cerevisiae* *SPR1* (GenBank NO. NM_001183609)** homologous DNA fragments.

| Primers | Oligonucleotide sequences (5'-3') |
| --- | --- |
| SPR_1 | GCCTCTCTGTTCATTATTTGTCCC |
| SPR_2 | TTGGCGGTCTCTGCCAGCTTAA |
| SPR_3 | GAATTCGGTACCGAAGCGGCGCGTCTGGTTCATT |
| SPR_4 | GCTAGCCGATGATGATGCATGCCCAATGACTATAAAGGCGTTCC |
| SPR_5 | ATGCATCATCATCGGCTAGCGTTAACGGATTTGATAACTCAGGGTTGA |
| SPR_6 | GAGCTCACCAATGCTTCTCGCTTACGGCATCTTTCCCCCATTGGC |
| SPR_7 | ACCAACCCCATTTAACCACTTT |
| SPR_8 | TTGTCTTTGGTCCAACTTCCATC |
| ADE2_SPR1 | TGTTATTGGTATCGGCATGCGGATCCATGCTAGCGATGCGGAATTGACTTTTTCTTG |
| ADE2_SPR2 | GTTAACGATCATCATCACTAGTGTGATCTTATGTATGAAATTCTTAA |

**Table S4. Primers used for cloning of *S. cerevisiae YIR007W*** (GenBank NO. NM_001179529) homologous DNA fragments.

| Primers | Oligonucleotide sequences (5'-3') |
| --- | --- |
| YIR_1 | CATGCTGGTCCAGGACAGTACG |
| YIR_2 | CAGCGTACAACAAGGTATGTAC |
| YIR_3 | GGTACCCTCACCAAGACGTCTGGTCTAGGTTTAGCGGTGGATCTG |
| YIR_4 | GCTAGCCGATGATGATGCATGCAGCTTTGAAATGCCGTTGGCGT |
| YIR_5 | ATGCATCATCATCGGCTAGCGTTAACCAAGTTTAGGGAATTCGATCAAG |
| YIR_6 | GAGCTCCTTCATTGTTCCAATTGTCACCCCATATATGTGAATTAA |
| YIR_7 | CTTGGTGGTTGTGAAGAACCCG |
| YIR_8 | TGCACGAGGGACTATCCAAATC |
| Ura3_YIR1 | CGGCATTTCAAAGCTGCATGCTCGCGAAGACTAGTTAAaCTTTTCAATTCATCATT |
| Ura3_YIR2 | TAAACTTGGTTAACGCTAGCTCTAGTGGGTAATAACTGATATAA |

**Table S5. Primers used for cloning of *S. cerevisiae* *PGM2* (GenBank NO. NM_001182605**) gene.

| Primers | Oligonucleotide sequences (5'-3') |
| --- | --- |
| PGM_Y1 | GAAGATCAACCAATCTTTCTCAG |
| PGM_Y2 | CATAACCATATGTCATTTCAAATTGAAACGGTTCCCACCAAACCTTATGAAGACCAAA |
| PGM_Y3 | CCAAACCTTATGAAGACCAAAAGCCTGGAACCTCTGGTTTGCGTAAGAAG |
| PGM_Y4 | AGACCATGGGCCTTGGCAACAC |
| PGM_Y5 | CAAGGCCCATGGTCTAAACTG |
| PGM_Y6 | ACACCCGATTTGGTAACATAAGCTCTCAATTGATCGACA |
| PGM_Y7 | AGCTTATGTTACCAAATCGGGTGT |
| PGM_Y8 | TTCGTTCTCGAGTCTAGATTAAGTACGAACCGTTGGTTC |
| PGM_Y9 | CTTCTTTACCGTTAATATTCATTG |

**Table S6. Primers used for cloning of *S. cerevisiae* *UGP1* (GenBank NO. NM_001179601**) gene.

| Primers | Oligonucleotide sequences (5'-3') |
| --- | --- |
| UGP1_1 | CTTCCACAGCAAGTGTACCATAAC |
| UGP1_2 | CATAACCATATGTCTACTAAAAAACATACTAAAACACATTCCACTTATGC |
| UGP1_3 | TCCCATTCCAAGGTGGTTCTTGAAGACTTCTCTACCAAA |
| UGP1_4 | AGAACCACCTTGGAATGGGA |
| UGP1_5 | TTGAAGGTCGACAGATCTGATTCTGATTCTGTTA |
| UGP1_6 | GAATCGGGATCCTTCAATCAATCCAGGTTCCC |
| UGP1_7 | CGCACCACTAGTTCAATGTTCCAAGATTTGCAAA |
| UGP1_8 | TAGAATAAATAACCTAACGGAGAATTG |


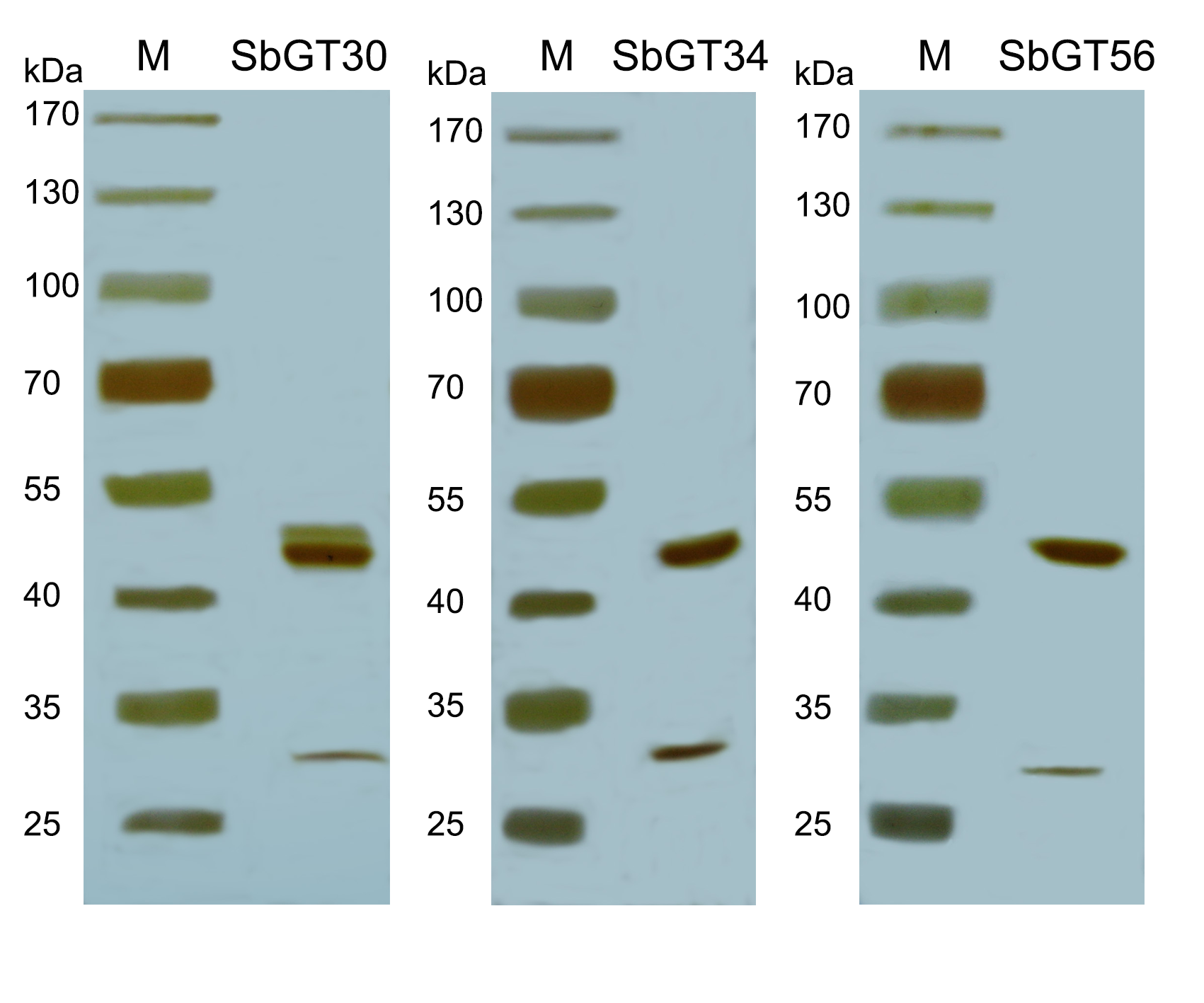
**Figure S1. SDS-PAGE analysis of recombinant SbGTs purified by affinity chromatography.** M, molecular weight marker; Lines SbGT30, SbGT34 and SbGT56: the purified recombinant protein SbGT30, SbGT34 and SbGT56, respectively.


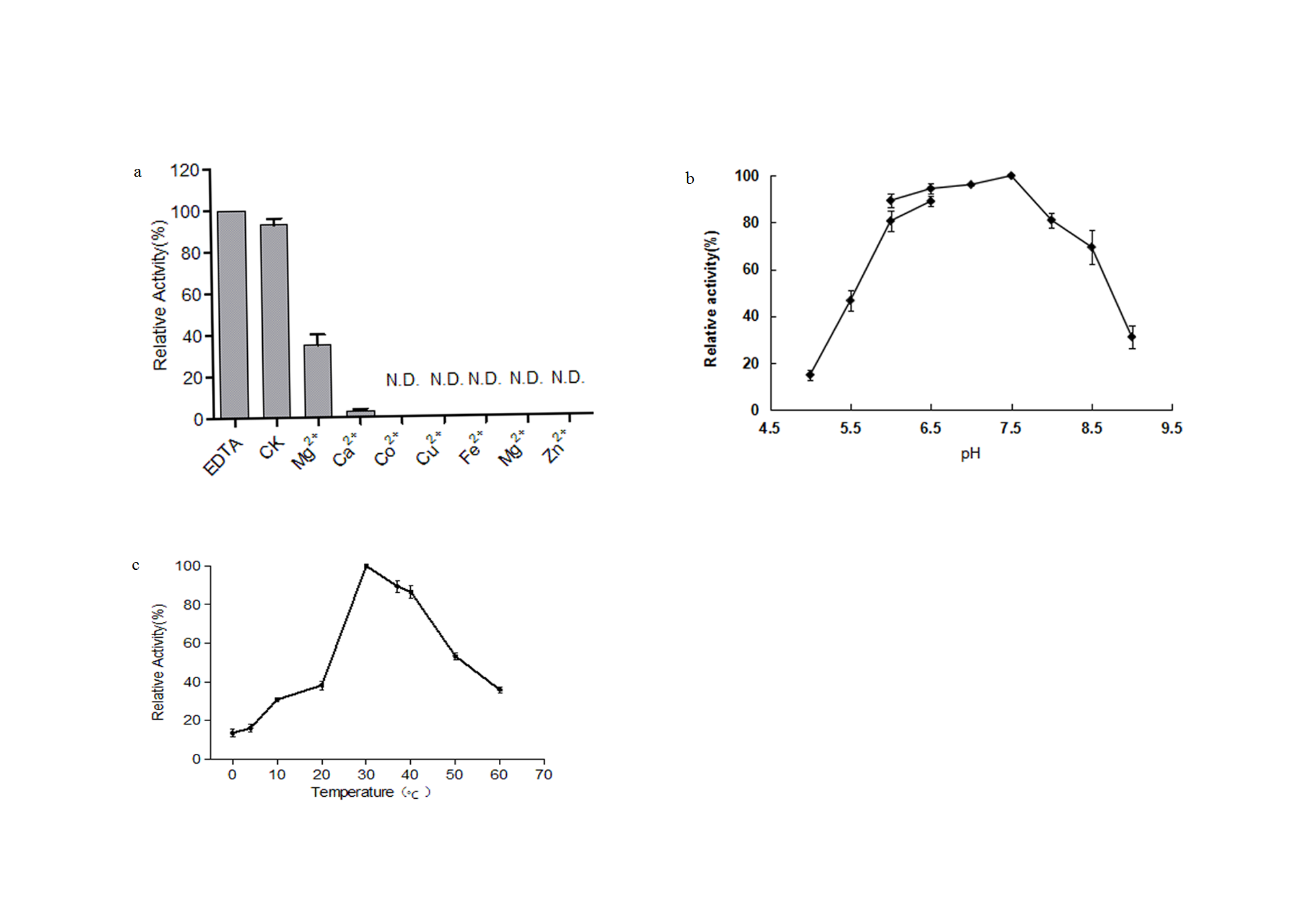


**Figure S2. Effects of various divalent metal ions (a), pH (b), and temperature (c) on enzyme activity of SbGT34.** UDP-Glu was used as a sugar donor and scutellarein was used as an aglycon acceptor. N.D. means not detected. The optimum temperature and pH of purified SbGT34 were 37℃ and 7.5, respectively. This enzyme was independent of metal ions.


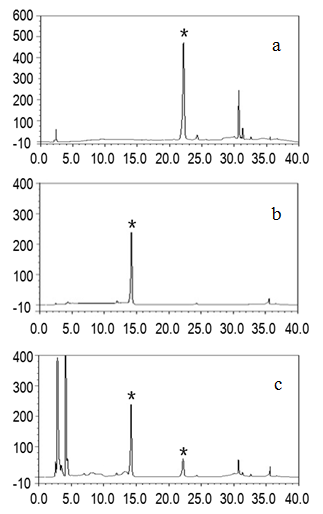


**Figure S3. Activity analysis of glycosidase in whole-cell *S. cerevisiae* using luteolin 7-*O*-glucoside as a substrate.** Standard luteolin (a), standard luteolin 7-*O*-glucoside (b), and reaction products of wild-type W303-1b with luteolin 7-*O*-glucoside as a substrate (c).


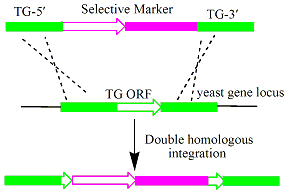


**Figure S4. Diagrammatic sketch of the knockout of glucosidase genes.** The TG-5' and TG-3' represent the homologous DNA fragments of the targeted β-glucosidase genes. Three auxotrophic selection markers *Trp1*, *Ade2* and *Ura3* were used to select the deletions of *EXG1*, *SPR1*, *YIR007W*, respectively, in this work. A double homologous integration replaced the glucosidase ORF in the genome.


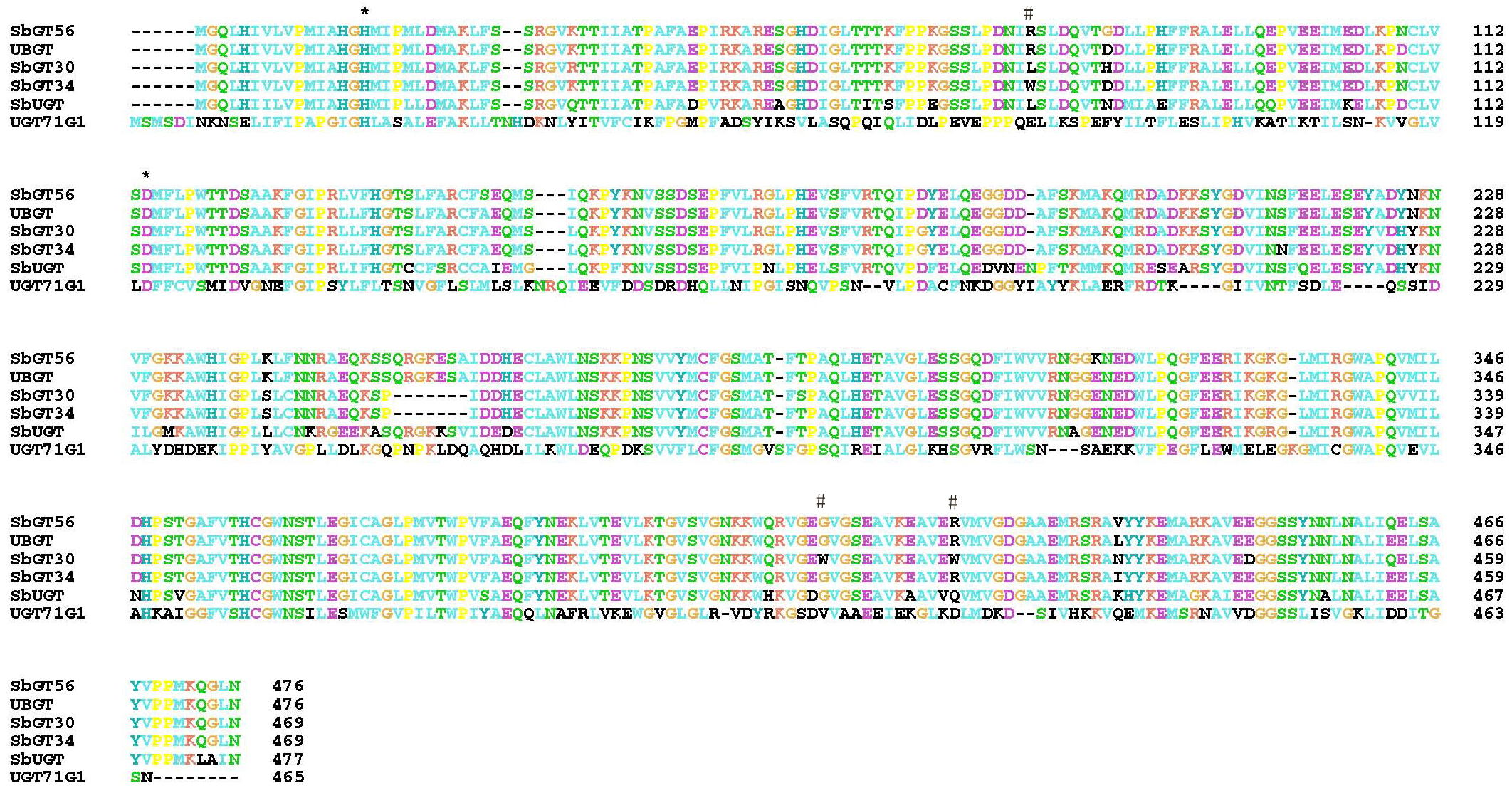


**Figure S5. A multiple alignment of the amino acid sequences of SbGT30, SbGT34, SbGT56, UBGT, SbUGT, and UGT71G1.** The enzyme active sites are marked with (*). The significantly different amino acid residues L75, W403 and W415 of SbGT30 are marked with (#). The natural deletion motif of SbGT34 and SbGT30 is underlined. The plant secondary product glycosyltransferase (PSPG) consensus sequence is highlighed with rectangle. Abbreviations: UBGT, *S. baicalensis* Georgi UDP-glucosyltransferase (AB031274); SbUGT, *S. barbata* UDP-glycosyltransferase barbata (GU339042); UGT71G1, *Medicago truncatula* UDP-glucosyltransferase (AY747627).


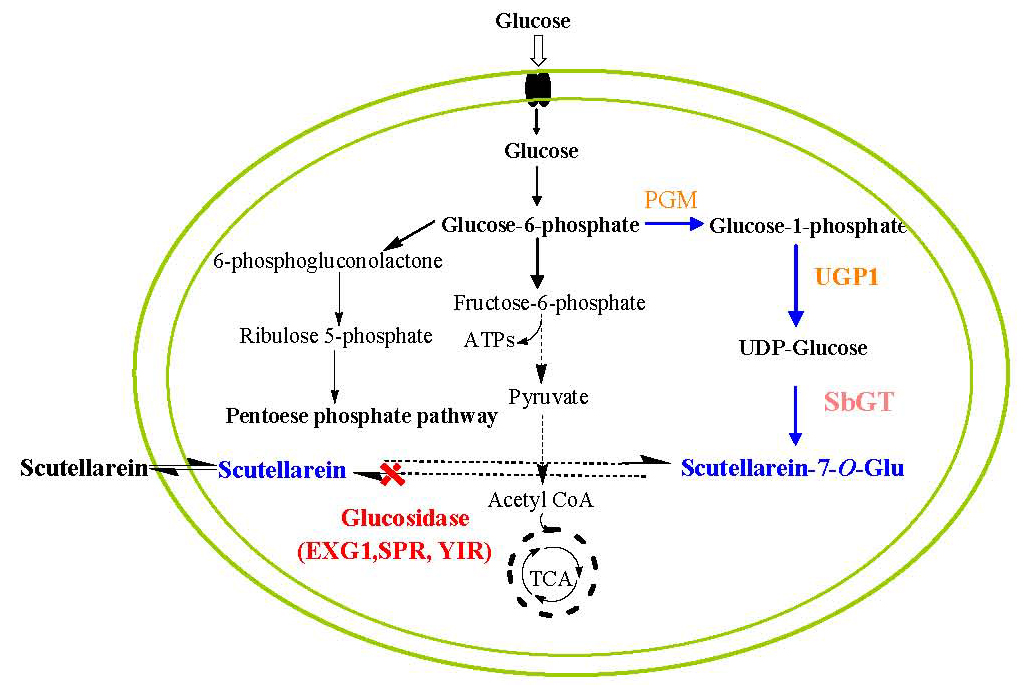


**Figure S6. Schematic diagram of biosynthetic pathway of scutellarein 7-*O*-glucoside.** *PGM*, phosphoglucomutase; *UGP1*, glucose-1-phosphate uridylyltransferase; *SbGT*, a SbGT34 clone of *S. baicalensis* Georgi UDP-glucosyltransferase expressed in this work. The dotted black arrow with marker “**×**” indicates the deletion of host β-glucosidase.

**Methods**

**Cloning of *S. baicalensis* SbGT cDNAs**

Total RNA was extracted from aseptic seedlings of *S. baicalensis* Georgi using an RNeasy Plant Mini Kit (TianGen Biotech, China), and cDNA was synthesized by reverse transcription using a GeneRacer™ RACE Kit (Invitrogen, USA) with a sequence-specific primer, UDPG5, designed according to the sequence of *S. baicalensis* Georgi *UBGT* (GenBank NO. AB031274). The full-length flavonoid glycosyltransferase DNA homologs were generated by nested-PCR with the primers UDPG1 to UDPG4 (Table S1), and cloned into a T-vector via TA cloning to generate pEZ-SbGT, verified by DNA sequencing. To prepare the recombinant enzymes, the verified cDNA fragments were amplified by PCR with the synthesized primers UDPG_E1 and UDPG_E1, and then ligated into a derivative (designated as pTWIN1B) of the plasmid pTWIN1 (NEB, Bio-Labs) constructed by replacing the C-intein region of the DNA fragment with a multiple cloning site (MCS) from the plasmid pET-28a (Novagen, Germany), which was amplified by PCR using the primers Twin1-B1 and Twin1-B2.

To construct yeast expression vector of SbGT34 clone, the SbGT34 encoding-DNA fragment was obtained by PCR using pEZ-SbGT34 as a template and primers SBUGT_N1 and SBUGT_X1. The obtained SbGT34 DNA fragment cleavaged with *Nde* I and *Xba* I was inserted into plasmid pδGAPg [1], generating plasmid pδGAPg-SbGT34.

**Construction of homologous integration vector of *S. cerevisiae EXG1* gene**

To functionally delete endogenous *EXG1* gene (GenBank No. M34341) in *S. cerevisiae*, two homologous DNA fragments of *EXG1* gene were amplified by Nested-PCR with the synthesized primers (Table S2).

5'-terminal DNA fragment F1 of *EXG1* was amplified by Nested-PCR. Primers EXG1_1 and EXG1_8 were used for the first-round amplification with the template of *S. cerevisiae* W303-1b genomic DNA, then fragment F1 was obtained by Nested-PCR from the first-round PCR product with primers EXG1_2 and EXG1_4. 3'-terminal DNA fragment F2 was obtained by Nested-PCR from the first-round PCR product with primers EXG1_5 and EXG1_7. A further complementary reaction between F1 and F2 fragments was carried out (95℃，5 min；95℃，50 s；43℃, 60 s；72℃，1 min，7 cycles；72℃，10 min；4℃，10 min). Then the recombinant homologous *EXG1* fragment of the expected size (EXG36, 1009 bp) was obtained from the product of the complementary reaction by PCR with primers EXG1_3 and EXG1_6. The resulting EXG36 fragment was cloned into a T-vector via TA cloning to generate pEZ-EXG36 and confirmed by DNA sequencing.

To select positive strain after deletion of *EXG1* gene, the auxotrophic marker *TRP1* was used. The full-length *TRP1* fragment was amplified from the genomic DNA of wild-type *S. cerevisiae* with primers Trp_EXG1 and Trp_EXG2. At last, the obtained fragment *Trp1* digested with *Nhe* I and *Hpa* I was inserted into plasmid pEZ-EXG36 digested by *Nhe* I and *Hpa* I, generating plasmid pEZ-EXG36-TRP1 used for the deletion of *EXG1* gene.

**Construction of homologous integration vector of *S. cerevisiae SPR1* gene**

To functionally delete endogenous *SPR1* gene (GenBank No. NM_001183609) in *S. cerevisiae*, two homologous DNA fragments of *SPR1* gene were amplified by Nested-PCR with the synthesized primers (Table S3).

5'-terminal DNA fragment F1 of *SPR1* was amplified by Nested-PCR. Primers SPR1_1 and SPR1_8 were used for the first-round amplification with the genomic DNA of *S. cerevisiae* W303-1b, then fragment F1 was obtained by Nested-PCR from the first-round PCR product with primers SPR1_2 and SPR1_4. 3'-terminal DNA fragment F2 was obtained by Nested-PCR from the first-round PCR product with primers SPR1_5 and SPR1_7. A further complementary reaction between F1 and F2 fragments was run (95℃，5min；95℃，50s；43℃, 60 s；72℃，1 min，7cycles；72℃，10 min；4℃，10 min). Then the recombinant homologous *SPR1* fragment of the expected size (SPR36, 1056 bp) was obtained from the product of the complementary reaction by PCR with primers SPR1_3 and SPR1_6. The resulting fragment SPR36 was cloned into a T-vector via TA cloning to give pEZ-SPR36 and confirmed by DNA sequencing.

To select positive strain after deletion of *SPR1* gene, the auxotrophic marker *ADE2* was used. The full-length *ADE2* fragment was obtained from the genomic DNA of wild-type *S. cerevisiae* by PCR with primers ADE2_SPR1 and ADE2_SPR2. At last, the obtained fragment *ADE2* DNA fragment digested with *Sph* I and *Spe* I was inserted into plasmid pEZ-SPR36 digested by *Sph* I and *Nhe* I, generating plasmid pEZ-SPR36-ADE2 using for the deletion of *SPR1* gene.

**Construction of homologous integration vector of *S. cerevisiae YIR007W* gene**

To functionally delete endogenous *YIR007W* gene (GenBank No. NM_001179529) in *S. cerevisiae*, two homologous DNA fragments of *YIR007W* gene were amplified by Nested-PCR with the synthesized primers (Table S4).

5'-terminal DNA fragment F1 of *YIR007W* was amplified by Nested-PCR. Primers YIR1_1 and YIR_8 were used for the first-step amplification with the genomic DNA of *S. cerevisiae* W303-1b, then fragment F1 was obtained by Nested-PCR from the first-round PCR product with primers YIR1_2 and YIR1_4. 3'-terminal DNA fragment F2 was obtained by Nested-PCR from the first-round PCR product with primers YIR1_5 and YIR1_7. A further complementary reaction between F1 and F2 fragments was carried out (95℃，5min；95℃，50s；43℃, 60 s；72℃，1 min，7cycles；72℃，10 min；4℃，10 min). Then the recombinant homologous *YIR007W* fragment of the expected size (YIR36, 1050 bp) was obtained from the product of the complementary reaction product by PCR with primers YIR1_3 and YIR1_6. The resulting fragment YIR36 was cloned into a T-vector via TA cloning to generate pEZ-YIR36 and confirmed by DNA sequencing.

To select positive strain after deletion of *YIR007W* gene, the auxotrophic marker *URA3* was used. The full-length *URA3* fragment was obtained from the genomic DNA of wild-type *S. cerevisiae* by PCR with primers URA3_YIR1 and URA3_YIR2. At last, the obtained fragment *URA3* DNA fragment digested with *Sph* I and *Nhe* I was inserted into plasmid pEZ-YIR36, generating plasmid pEZ-YIR36-URA3 used for the deletion of *YIR1* gene.

**Cloning of *S. cerevisiae PGM2* gene encoding phosphoglucomutase.**

Based on the encoding sequence of *S. cerevisiae* *PGM2* (GenBank No. NM_001182605), the synthesized primers (Table S5) were used to clone the whole DNA fragment of *PGM2* which was divided into three parts for DNA fragment assembly.

3'-terminal DNA fragment F1 of *PGM2* was amplified by Nested-PCR. Primers PGM_Y1 and PGM_Y9 were used for the first-round amplification with the genomic DNA of *S. cerevisiae* W303-1b as template. Two parts of F1 fragment was obtained by Nested-PCR from the first-round PCR product with two pairs of primers PGM_Y5/PGM_Y6 and PGM_Y7/PGM_Y8, respectively, and then a complementary reaction between the two resulting fragments was carried out (95℃，5min；95℃，50s；43℃, 60 s；72℃，45 s，7cycles；72℃，10 min；4℃，10 min). The 3'-terminal DNA fragment F1 (PGM58) was amplified from the product of the complementary reaction with primers PGM_Y5 and PGM_Y8. The resulting PGM58 fragment was cloned into a T-vector via TA cloning to generate pEZ-PGM58, and confirmed by DNA sequencing.

5'-terminal DNA fragment F2 (PGM34) was obtained by Nested-PCR from the first-round PCR product (PGM_Y1 and PGM_Y9) with primers PGM_Y3 and PGM_Y4. The resulting PGM34 fragment was cloned into a T-vector via TA cloning to generate pEZ-PGM34, and confirmed by DNA sequencing. At last, plasmid pEZ-PGM38 was obtained using site *Nco* I through splicing and ligation of two DNA fragments forming a read frame. The full-length *PGM2* fragment of the expected size (PGM28, 1710 bp) was obtained by PCR using pEZ-PGM38 as template and primers PGM_Y2 and PGM_Y8. The obtained PGM28 fragment digested with *Nde* I and *Xba* I was inserted into plasmid pδGAPg [1] digested by *Nde* I and *Nhe* I, generating plasmid pδGAPg-PGM2.

**Cloning of S*. cerevisiae UGP1* gene encoding UTP glucose -1- phosphate uridylyltransferase.**

Based on the encoding sequence of *S. cerevisiae* *UGP1* (GenBank No. NM_001179601), the synthesized primers (Table S6) were used to clone the whole DNA fragment of *UGP1*.

DNA fragment of *UGP1* was amplified by Nested-PCR. Primers UGP1_1 and UGP1_8 were used for the first-round amplification with the template of *S. cerevisiae* W303-1b genomic DNA. 5'-terminal DNA fragment of *UGP1* was obtained from the first-round PCR product as a template using two pairs of primers UGP1_2/UGP1_3 and UGP1_4/UGP1_5, respectively. The resulting two parts were spliced through overlapping PCR (95℃，5 min；95℃，50 s；43℃, 50 s；72℃，45 s，7 cycles；72℃，10 min；4℃，10 min), of which the product was used as template for amplification of 5'-terminal DNA fragment with primers UGP1_2 and UGP1_5. The resulting fragment UGP25 was cloned into a T-vector via TA cloning to generate pEZ-UGP25, and confirmed by DNA sequencing.

3'-terminal DNA fragment of *UGP1* was obtained by Nested-PCR from the first-round PCR product with primers UGP1_6 and UGP1_7. The resulting fragment UGP67 was cloned into a T-vector via TA cloning to generate pEZ-UGP67, and confirmed by DNA sequencing. At last, fragment UGP67 obtained by digestion of plasmid pEZ-UGP67 with *Bam*H I and *Xba* I was inserted into plasmid pEZ-UGP25 digested by *Bgl* II and *Xba* I, generating plasmid pEZ-UGP27. The full-length *UGP1* fragment of the expected size (UGP27, 1500 bp) was obtained by PCR using pEZ-UGP27 as template and primers UGP1_2/ and UGP1_7. The obtained fragment UGP27 digested with *Nde* I and *Spe* I was inserted into plasmid pδGAPh [1] digested by *Nde* I and *Nhe* I, generating plasmid pδGAPh-UGP1.

**Measurement of glucosidase activity in *S. cerevisiae***

The hydrolytic activity of glucosidases in *S. cerevisia*e was measured by directly adding luteolin 7-*O*-glucoside in a yeast culture solution. The wild-type yeast strain W303-1b was grown at 30°C in YPD medium for 24 h, and the culture was then inoculated into fresh SC medium at 1% as the second seed, and the cells were grown at 30°C for approximately 10 h. Then, the OD_600_ of the second seed was adjusted to 1.0 using fresh SC medium, and 0.2 mM luteolin 7-*O*-glucoside was added to the medium. The mixture was incubated at 30°C for 6 h, and 1 ml of reaction medium in a 5-ml reaction flasks was dried and was extracted using 500 μL of methanol three times. The methanol extracts were evaporated and then re-dissolved in 1 mL of methanol. The results were identified by HPLC analysis.

**Results**

**Identification of glucosylation products**

Scutellarein 7-*O*-glucoside: ESI-MS: m/z = 471 [M+Na]^+^, ESI-MS/MS: m/z = 287 [M+H-C_6_H_10_O_5_]^+^; ^1^H NMR (DMSO-d6, 500 MHz) δ = 7.01 (s, 1H; H-3), 6.83 (s, 1H; H-8), 7.94 (d, J = 10.0 Hz, 2H; H-2', H-6'), 7.95 (, J = 10.0 Hz, 2H; H-3', H-5'), 4.99 (d, J = 10.0 Hz, 1H; H-1"), 3.27–3.77(m, 5H; H-3", H-4", H-5", H-6"), 4.25 (d, J = 10.0 Hz, 1H; H-2"); ^13^C NMR (DMSO-d6, 125 MHz) δ = 164.5 (C-2), 102.9 (C-3), 182.9 (C-4), 147.0 (C-5), 130.9 (C-6), 151.8 (C-7), 94.5 (C-8), 149.5 (C-9), 106.3 (C-10), 121.7 (C-1'), 128.9 (C-2', C-5'), 116.5 (C-3', C-5'), 161.8 (C-4'), 101.3 (C-1"), 73.6 (C-2"), 76.3 (C-3"), 70.1 (C-4"), 77.8 (C-5"), 61.7 (C-6").

**Reference**

1. Tang L, Wang W, Zhou W, Cheng K, Yang Y, Liu M, et al. Three-pathway combination for glutathione biosynthesis in *Saccharomyces cerevisiae*. Microb Cell Fact. 2015; 14:139.
